# Reliability and Validity of the Colombian Version of the Revised Sociosexual Orientation Inventory

**DOI:** 10.1007/s10508-022-02402-8

**Published:** 2022-09-12

**Authors:** Duban Romero, Moisés Mebarak, Anthony Millán, Juan Camilo Tovar-Castro, Martha Martinez, David L. Rodrigues

**Affiliations:** 1 Department of Psychology, Universidad del Norte, Km.5 Vía Puerto Colombia, Barranquilla, 081007 Colombia; 2 Department of Psychology, Universidad de los Andes, Bogotá, Colombia; 3 Department of Psychology, Universidad Simón Bolivar, Barranquilla, Colombia; 4 Iscte-Instituto Universitário de Lisboa, CIS-Iscte, Lisbon, Portugal

**Keywords:** Sociosexuality, Sociosexual Orientation Inventory-Revised (SOI-R), Factor analysis, Colombia

## Abstract

Sociosexuality refers to an individual’s disposition to have casual sex without establishing affective bonds and has been widely studied worldwide using the Revised Sociosexual Orientation Inventory (SOI-R; Penke & Asendorpf, 2008). Despite its many validations in different cultural contexts, no psychometric analyses of this instrument have been conducted in Spanish-speaking Latin American countries. To address this gap in the literature, we examined the psychometric properties of the SOI-R in Colombia. In a cross-sectional study with a large sample of participants (*N* = 812; 64% women), we conducted exploratory and confirmatory factor analyses to identify different factor structures and determine which had the best fit for our sample and examined the reliability of the scale. Results showed that a three-factor structure, with sociosexual behaviors, attitudes, and desire as first-order factors, and global sociosexuality as a second-order factor, had the best fit indexes. Each factor presented good reliability indexes. Replicating already established gender differences, we also found that men scored higher on each factor when compared to women. These findings show that the SOI-R is a reliable and valid instrument to assess sociosexuality in countries where sociosexuality research is underrepresented.

## Introduction

Animal behavioral studies relate sociosexuality to sexual behaviors that occur in non-sexual contexts and facilitate social interaction, thus implying an adaptive role for individuals in a species (e.g., Cross et al., 2021; Lilley et al., 2020). Since Kinsey et al. (1948) coined the term sociosexual behaviors in studies of human sexuality, psychologists have been using sociosexuality to indicate an individual disposition to have casual sex without establishing affective bonds. In their seminal work focused on sociosexuality in humans, Simpson and Gangestad (1991) conceptualized sociosexuality as a unidimensional construct reflecting past behaviors (e.g., frequency of sex in the past month), behavioral intentions (e.g., number of casual partners foreseen for the future), and attitudes toward casual sex. The authors developed and validated the Sociosexual Orientation Inventory (SOI) and found that people with an unrestricted sociosexual orientation were more likely to have sex with extradyadic partners and had fewer expressions of love and commitment to the romantic partner when compared to people with a restricted sociosexual orientation.

This conceptualization was later revised, based on psychometric evidence that sociosexuality was best represented as a multidimensional construct (for discussions, see Jackson & Kirkpatrick, 2007; Penke & Asendorpf, 2008; Webster & Bryan, 2007). For example, Webster and Bryan (2007) found better fit indexes for a two-factor model that differentiated between behavioral and attitudinal aspects of sociosexuality (for similar results, see Banai & Pavela, 2015). Further detailing the sociosexuality construct, Penke and Asendorpf (2008) reasoned that the original sociosexuality conceptualization did not consider evolutionary psychological mechanisms that could influence mating strategies. Hence, the authors suggested adding sociosexual desire to the conceptualization of sociosexuality and proposed the Revised SOI (SOI-R).

This psychometrically sound instrument assesses sociosexual behaviors, i.e., frequency of casual sex activities, sociosexual attitudes, i.e., cognitive evaluations of casual sex, and sociosexual desire, i.e., interest in having sex without commitment motivated by sexual fantasies and arousal. The SOI-R has become one of the most widely used instruments to assess and explore individual differences in sociosexuality and has been validated in different socio-cultural contexts, including Hungary (Meskó et al., 2014), Portugal (Neto, 2016; Rodrigues & Lopes, 2017), Spain (Barrada et al., 2018), Brazil (Nascimento et al., 2018), and Ghana (Koomson & Teye-Kwadjo, 2021).

These studies show that the SOI-R has adequate reliability across different countries and, aligned with the revision proposed by Penke and Asendorpf (2008), has a three-factor structure. Hence, most researchers compute the score of each factor separately proceeding to the analyses (e.g., Barrada et al., 2018; Botnen et al., 2018). However, other authors compute scores for each factor and a score for global sociosexuality (e.g., Bártová et al., 2020; Gomula et al., 2014; Howell et al., 2012; Randler et al., 2016; Rodrigues & Lopes, 2017), and others only compute an overall sociosexuality score (e.g., Brown & Sacco, 2017). From our perspective, this may have implications for conclusions, comparisons across studies, and generalizations, especially considering that the SOI-R was conceptualized under the premise that sociosexuality is more than a global construct (Penke & Asendorpf, 2008).

Research has consistently shown that men tend to have a more unrestricted sociosexual orientation when compared to women (e.g., Lippa, 2009; Schmitt, 2005). Despite these gender differences, research has also shown that sociosexual orientation provides insight into various aspects of individuals' sexual behavior. For example, people with unrestricted sociosexuality tend to have lower sexual disgust, even if they have a different level of moral and pathological disgust, which can arguably help optimize their mating strategies (Al-Shawaf et al., 2015; Banai et al., 2021), and higher voyeuristic and exhibitionist behaviors (Thomas et al., 2021). These individuals are also more likely to engage in a wider array of online and offline sexual behaviors (Martins et al., 2016; Rodrigues et al., 2017; Weiser et al., 2018; Zheng & Zheng, 2014) and have more risky sexual behaviors (Lopez et al., 2021). In relationships, people with more unrestricted sociosexuality tend to have reported less quality and more dissatisfaction in their relationships (Hall & Pichon, 2014; Rodrigues et al., 2017), engage in more extradyadic behaviors (especially men) when they have more potential partners available (Arnocky et al., 2016; Weiser et al., 2018), and have a lower predisposition for parenting (Valentova et al., 2019, 2020).

The few studies that assessed sociosexuality in Spanish-speaking countries have shown that people with unrestricted sociosexuality tend to masturbate and have sexual contact with regular and casual partners more often, endorse fewer myths and taboos related to sexual scripts, and are more likely to engage in extradyadic sex when they feel dissatisfied with their partner’s sexual performance (Pérez & Díaz-Loving, 2013; Rodríguez & Loving, 2011; Valdebenito et al., 2018). However, researchers in these countries have failed to properly examine the psychometric characteristics of SOI-R (except for Spain; Barrada et al., 2018). Research in Colombia examining sociosexuality and how it might shape sexual activity and sexual risk-taking is still scarce (for an exception with a limited sample of Colombian people, see Marcinkowska et al., 2019). Hence, our main goal is to examine the psychometric properties of the Spanish version of the SOI-R in a large Colombian sample, using exploratory and confirmatory factor analyses. It expects to provide researchers with objective indicators of the reliability and validity of the SOI-R to adequately assess sociosexuality in a Spanish-speaking Latin American countries, and therefore extend the cross-cultural generalizability of the findings when using this instrument. Hence, this study aims to establish the reliability and validity of the Spanish version of the SOI-R in a sample of Colombian adults, which will allow us to have a reliable psychometric instrument to objectively measure sociosexuality in this region.

## Method

### Participants

A total of 1033 people responded to the online survey, of which 166 abandoned the survey before completion, and 55 did not respond correctly to the control item. The final sample included 812 participants (64% women) with ages between 18 and 60 (*M* = 22.99, *SD* = 7.24, Q3 = 23 years). The sample mostly included heterosexual people (87%), college students (75.1%), and people living in Colombia’s Caribbean coast (96%). Nearly half of our participants indicated to be single (46.7%), whereas 41.7% were currently in a romantic relationship.

### Measures

### Demographic Information

We asked participants to indicate their age in years (open-ended question), biological sex (male, female, and other), sexual orientation (heterosexual, homosexual, bisexual, and other), completed educational level (high school, college, bachelor, graduate studies, and no studies), marital status (single, dating, common-law marriage, and married), and area of residence (in which department of Colombia do you live?).

### Sociosexual Orientation

The SOI-R is a self-report instrument developed by Penke and Asendorpf (2008) that assesses sociosexual behavior (three items; e.g., “How many partners have you had sex with in your life?”), sociosexual attitudes (three items; e.g., “Sex without love is okay”), and sociosexual desire (three items; e.g., “How often do you experience sexual arousal when you are in contact with someone you are not in a committed romantic relationship?”). Responses to the items can be given in 9-point or 5-point rating scales, with evidence showing similar psychometric properties between versions. In this study, we validated the SOI-R with a 5-point response scale (response labels differed according to the item) to facilitate responses among people who are not familiar with psychological tests (e.g., non-student population). For ease of adaptation, we used the Spanish version of the SOI-R (Barrada et al., 2018). In this version, sociosexual behavior is addressed with specific reference to penetrative sex (e.g., “How many partners have you had penetrative sex with in your life?”).

### Procedure

This research project was approved by the Ethical Committee at Universidad del Norte, Colombia (No. 185/2019). Data were collected through an online survey via Google Forms from April 2019 to May 2020. Prospective participants were recruited through Facebook Ads. Specifically, people who were at least 18 years old, sexually active, and lived in Colombia were invited to participate. The first page of the web survey included contact information for psychological care centers in case participants experienced any adverse emotional responses during their participation. Participants had to agree to the terms and conditions of the study before proceeding. To ensure the quality of data, we included one attention-check item (Please check the option “Completely agree”). Responses of participants who did not answer correctly to the control item were excluded. We asked about the region of Colombia where participants were located to ensure just people living in Colombia responded to the measures. The survey included other measures that were not relevant for this study.

### Data Analysis

The complete dataset for this study is available on Open Science Framework. We followed the guidelines of the two-step method (Anderson & Gerbing, 1988; Hair et al., 2014; Lloret-Segura et al., 2014), which consisted in splitting the sample into two random subsamples. This method has been widely used in psychometric studies (e.g., Gravini-Donado et al., 2019; Lange et al., 2013, 2016; Restrepo et al., 2021). We then computed Exploratory Factor Analyses (EFA) in the first subsample (*n* = 405), using the “fa” function of the “psych” package (Revelle, 2020) in version R 4.0.2 (R Core Team, 2020). We used the main axis method with promax rotation due to the expected correlation between sociosexuality factors (i.e., obliquity between factors). To assess the appropriateness of implementing the EFA from Pearson and Polychoric correlation matrices, we examined the Kaiser–Meyer–Olkin test (KMO) and Bartlett’s test of sphericity. The EFA allowed us to test different factorial structures of the Colombian version of the SOI-R, based on eigenvalues, a scree plot, and a parallel analysis (for a discussion, see Lloret-Segura et al., 2014).

In the second subsample (*n* = 407), we computed Confirmatory Factor Analyses (CFA) using the “lavaan” package functions in R (Rosseel, 2012). We also used CFA to evaluate each factorial model identified by the EFA. We calculated absolute, incremental, and parsimony fit indexes for each model. The absolute fit indices were χ^2^, normed χ^2^, Goodness of Fit Index (GFI), Approximation Mean Square Error (RMSEA), Relative Non Centrality Index (RNI), Expected Cross Validation Index (ECVI), and Root Mean Square Residue (RMSR). The incremental adjustment indexes were the Goodness of Fit Index (AGFI), Tucker-Lewis Index (TLI), Normalized Fit Index (NFI), and Comparative Fit Index (CFI). The parsimony adjustment measures were Parsimony Adjustment Normalized Index (PNFI) and Parsimony Adjustment Goodness Index (PGFI). Although psychometric studies show that the SOI-R has a three-factor structure, the scoring method varies in some studies. Thus, we also compared fit indexes of models based on such scoring methods.

We then examined the reliability of each factor in the final model using the Omega coefficient (McDonald, 1999). This statistic is recommended to assess the reliability of multidimensional scales composed of ordinal items in contrast to Cronbach's alpha which is more appropriate for unidimensional continuous measures consisting of fewer items (see Hayes & Coutts, 2020). Lastly, we replicated widely established gender differences, by comparing scores using Welch’s *t*-test and Cohen’s *d* for effect size.

## Results

### Exploratory Factorial Analysis

The Pearson correlation matrix (KMO = 0.85, Bartlett’s test of sphericity: χ^2^(36) = 2162.8, *p* < .001) and the polychoric correlation matrix (KMO = 0.85, Bartlett’s test of sphericity: χ^2^(36) = 682.6, *p* < .001) showed the adequacy of computing EFA in our subsample. Based on the parallel analysis criterion, the polychoric correlation matrix suggested a model with two factors (66.8% cumulative variance) that combined sociosexual attitudes and sociosexual desire and kept sociosexual behavior as an independent factor (Model 1). Based on the eigenvalue’s criterion, both correlation matrices (Pearson = 64.8% cumulative variance; polychoric = 72.6% cumulative variance) suggested a model with three factors (Model 2). This model replicated those identified by previous studies (Barrada et al., 2018; Meskó et al., 2014; Nascimento et al., 2018; Neto, 2016; Penke & Asendorpf, 2008; Rodrigues & Lopes, 2017). Based on the scree plot criterion, each correlation matrix suggested two other models. However, these were discarded from subsequent analyses because neither model agglomerated enough items into each factor.

### Confirmatory Factorial Analysis

**Fig. 1:**
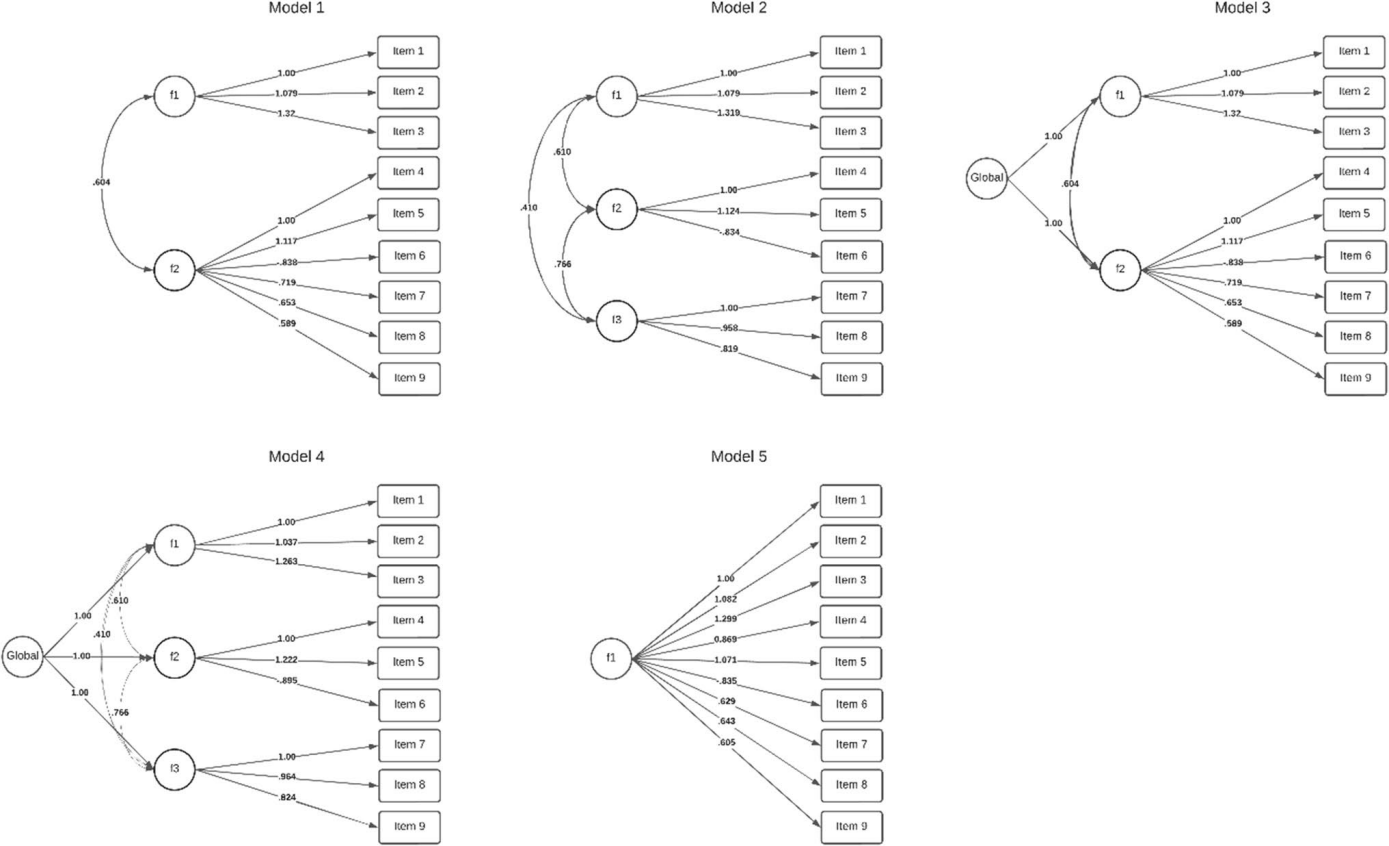
Tested models

To these analyses, we added a model that included a second-order factor for the two-factor model (Model 3), a model that included a second-order factor for the three-factor model (Model 4), and a one-factor model (Model 5). Figure 1 depicts each of the tested models and Table 1 summarizes the fit index of each model. As can be seen, Models 2 and 4 had the best fit indexes, whereas Model 5 had the worst fit indexes. Given that Model 4 included sociosexuality as a global score we decided to choose this as the final factor structure. Table 2 summarizes the matrix of rotated loads and the descriptive statistics of each item.

**Table 1:** Absolute, incremental and parsimony fit indexes by model

	χ^2^	*df*	CFI	TLI	NFI	PNFI	RNI	RMSEA	SRMR	GFI	AGFI	PGFI	ECVI
Model 1	321.35^***^	26	.86	.81	.85	.61	.86	.17	.09	.82	.70	.48	.89
Model 2	38.35^*^	24	.99	.99	.98	.65	.99	.04	.03	.98	.96	.52	.20
Model 3	321.35^***^	26	.86	.81	.85	.61	.86	.18	.09	.82	.69	.48	.88
Model 4	65.83^***^	26	.98	.97	.97	.70	.98	.06	.08	.96	.94	.56	.25
Model 5	851.52^***^	27	.62	.49	.61	.46	.62	.27	.17	.61	.35	.37	2.18

***p < .001; **p < .010; *p < .050

**Table 2:** Matrix of rotated loads, descriptive statistics and measure of sample adequacy

	SOI-R Factors			Descriptive statistics		MSA
	Sociosexual behavior	Sociosexual attitude	Sociosexual desire	*M*	SD	
Item 1	.86			3.35	1.09	.83
Item 2	.81			2.21	1.16	.87
Item 3	.90			2.33	1.25	.81
Item 4		.87		3.07	1.45	.84
Item 5		.87		2.69	1.53	.84
Item 6		.61		3.21	1.45	.92
Item 7			.80	2.73	1.25	.86
Item 8			.92	2.48	1.24	.83
Item 9			.72	2.17	1.23	.90
Reliability	.90	.85	.87	–	–	–

*Note:* MSA = Measure of Sampling Adequacy; Reliability calculated using the Omega coefficient. Absolute range of SOI-R items = 1–5

### Reliability and Correlations Between Factors

Results showed an adequate reliability for the overall score of sociosexuality, *Ω* = 0.94, as well as for each factor separately (see Table 2). As expected, overall sociosexuality was positively associated with sociosexual behavior, *r*(810) = 0.76, *p* < .001, sociosexual attitudes, *r*(810) = 0.85, *p* < .001, and sociosexual desire, *r*(810) = 0.78 *p* < .001. Sociosexual behavior was associated with more sociosexual attitude, *r*(810) = 0.49, *p* < .001, more sociosexual desire, *r* (810) = 0.39, *p* < .001, and sociosexual attitude had a positive association with sociosexual desire, *r*(810) = 0.53, *p* < .001.

### Gender Differences in Sociosexuality

Table 3 summarizes the descriptive statistics of men and women for overall sociosexuality and for each factor separately. Men had higher scores than women in the overall score, *t*(621) = 13.70, *p* < .001, *d* = 0.99, sociosexual behavior, *t*(536) = 10.31, *p* < .001, *d* = 0.77, sociosexual attitude, *t*(653) = 10.94, *p* < .001, *d* = 0.79, and sociosexual desire, *t*(542) = 9.89, *p* < .001, *d* = 0.74.

**Table 3:** SOI-R descriptive statistics according to gender

	Overall sample (*n* = 812)	Women (*n* = 519)	Men (*n* = 293)
	*M* (SD)	*M* (SD)	*M* (SD)
Overall sociosexuality^a^	24.15 (1.17)	21.50 (1.17)	28.90 (1.17)
Sociosexual behavior^b^	7.82 (3.22)	6.97 (2.84)	9.33 (3.29)
Sociosexual attitudes^b^	9.03 (3.84)	8.03 (3.71)	10.80 (3.39)
Sociosexual desire^b^	7.29 (3.25)	6.46 (2.91)	8.76 (3.32)

Absolute range: 9–45^a^, 3–15^b^

## Discussion

We conducted a study with a large sample of Colombian adults to examine the psychometric properties of the Colombian version of the SOI-R. Overall, results replicated previous studies (Barrada et al., 2018; Meskó et al., 2014; Nascimento et al., 2018; Neto, 2016; Penke & Asendorpf, 2008; Rodrigues & Lopes, 2017), such that a model with three intercorrelated factors and a second-order factor was deemed the most adequate for our sample. Likewise, we found adequate indexes of internal consistency for the overall scale and each of its factors. These findings showed for the first time that the SOI-R is a reliable and valid instrument to be used in Colombia, thus providing evidence for support of its use when examining how sociosexuality can shape different processes in sexuality and sexual behavior in this context. More importantly, these findings also replicate Barrada et al. (2018), lending further support to the use of the SOI-R in Colombia (and other Latin-American Spanish-speaking countries), therefore using this instrument for cross-culturally comparison is possible. We also found evidence of construct validity, showing that the SOI-R items grouped into a factor structure similar to that found in other psychometric studies (Barrada et al., 2018; Meskó et al., 2014; Nascimento et al., 2018; Rodrigues & Lopes, 2017). In this study, the first- and second-order three-dimensional models showed adequate goodness of fit for what it is shown when calculating factorial scores for both the three dimensions of sociosexuality and on a general level. Lastly, our results replicated gender differences widely established cross-culturally (Lippa, 2009; Schmitt, 2005), including in Latin American countries (Pérez & Díaz-Loving, 2013).

The main strength of this study is that we evaluated the psychometric properties of SOI-R in a Spanish-speaking Latin American country for the first time, which facilitates future research in several countries of this area. Furthermore, we implemented statistical methods that made it possible to test different factor models identified through empirical evidence and past research. This procedure significantly reduced factorial indeterminacy, confirmatory, and chance capitalization biases. Future research should seek to replicate and extend our findings by using more diverse samples and examine how differences in sociosexuality relate to different sexual behaviors (e.g., Martins et al., 2016; Rodrigues et al., 2017) and sexual health decision-making (e.g., condom use and testing for sexually transmitted infections; Rodrigues et al., 2019).

In conclusion, the SOI-R is an instrument widely used in human sexuality studies to measure an individual’s predisposition toward casual sex. Our findings on a Colombian sample showed that this instrument has adequate reliability and validity. This study opened the door for other Latin American countries to pursue studies examining the correlates and implications of sociosexuality.
